# Balancing on the edge of age: neuroendocrine, mental health and functional fitness correlates of fear of falling in older women

**DOI:** 10.1016/j.clinsp.2025.100792

**Published:** 2025-10-15

**Authors:** Rafael N. Rodrigues, Dina Maria Mamede Pereira, Sónia Brito-Costa, António Felipe Souza-Gomes, Natália Oiring de Castro Cezar, Júlia Maria D’Andréa Greve, Angelica Castilho Alonso, Felipe Santos Marques, José Pedro Ferreira, Ana Maria Teixeira, Guilherme Eustáquio Furtado

**Affiliations:** a CIPER, Faculty of Sport Sciences and Physical Education, University of Coimbra, Coimbra, Portugal; b Polytechnic University of Coimbra, Coimbra, Portugal; c InED ‒ Center for Research and Innovation in Education, Coimbra Education School, Polytechnic University of Porto, Porto, Portugal; d Laboratory of Inflammation and Exercise Immunology, Universidade Federal de Ouro Preto, Ouro Preto, MG, Brazil; e Biomedical and Health Sciences, Universidade Estatual de Minas Gerais, Passos, MG, Brazil; f Movement Study Laboratory, Instituto de Ortopedia e Traumatologia do Hospital das Clínicas (IOT-HC), Faculdade de Medicina da Universidade de São Paulo (FMUSP), São Paulo, SP, Brazil; g Graduate Program in Aging Sciences at Universidade São Judas Tadeu (USJT), São Paulo, Brazil; h SPRINT ‒ Sport Physical Activity and Health Research & INnovation Center, Polytechnic University of Coimbra, Coimbra, Portugal

**Keywords:** Aging, Fall risk, Neuroendocrine system, Sex steroids hormones, Saliva, Stress, Brain health

## Abstract

**Introduction:**

Fear of Falling (FOF) is a common and natural behavior experienced by most of the elderly population during aging. It is thought to be caused by previous falls, dizziness, depression, and neuromotor impairments. However, the association between FOF and salivary biomarkers such as Cortisol (COR), Testosterone (TT), Dehydroepiandrosterone (DHEA), and alpha-amylase activities has not been consistently explored.

**Objective:**

To explore the association between FOF and general health status, Functional Fitness (FF), and salivary-related stress biomarkers in institutionalized older women.

**Materials and method:**

A total of 278 older women were assessed and grouped as having Higher (*n* = 176) or Lower FOF (*n* = 102), according to the Falls Efficacy Scale. Saliva samples were collected.

**Results:**

The Higher FOF group had superior scores in the depressive state and comorbidity scale (*p* ≤ 0.05). Participants with Lower FOF showed better FF scores. Salivary levels of cortisol were higher, and DHEA levels were lower in the High FOF group (*p* ≤ 0.05). Spearman's Rank test showed that FOF was inversely correlated with DHEA, alpha-amylase, and FF, whereas COR was directly associated with the FOF scale. Regression analysis indicated that functional fitness indicators were significantly associated with variations in FOF. However, after adjusting for covariates, the influence of physical-functional fitness was reduced, while depressive symptoms and DHEA levels remained statistically significant in the model, suggesting their stronger association with FOF. The observed associations between FOF and biomarkers, especially DHEA, add important evidence to the growing body of literature exploring the multifactorial nature of FOF. These findings contribute to a deeper understanding of the biological components involved and may help inform fall-risk monitoring strategies in institutionalized older populations.

## Highlights

• FOF relates to low fitness and altered biomarkers (↑cortisol, ↓DHEA).
• Depressive symptoms mediate the link between fitness and FOF.
• DHEA may serve as a biomarker for predicting FOF in older women.

## Introduction

Fear of Falling (FOF) is a common and natural behavior that most people experience during aging in varying degrees of severity.^1^ It can also be referred to as a phobia syndrome called basophobia and may be associated with astasia-abasia, or the fear of walking and/or even standing.^2^ Since then, FOF gained substantial attention as a health issue, specifically in the older adult population, who had fallen or not.^3^

The prevalence of FOF varies from 20 % to >80 % in community-dwelling older individuals, and this prevalence is greater than 50 % among adults living in social and health care centers.^4^ The FOF increases with age and is more prevalent in women, and other aspects can also contribute to increasing this prevalence, such as dizziness, health status, depression, and physical limitations.^5^ Based on these aspects, the risk of falls and FOF has a multifactorial origin.^6^

Many activities, such as regular exercise and social interactions, are avoided or restricted by older adults who are afraid of falling.^7^ Also, the increase of FOF in advanced aging was associated with a progressive functional decline, and consequently decreases the ability to perform instrumental and independent daily-life activities,^8^ which compromise physical activity levels, and may result in a vicious cycle.^9^

Psychological factors (i.e., depression, anxiety, stress) are also identified as main FOF risk factors, and have the potential to increase the risk of falls in older individuals due to their inverse interaction with physical and functional health components.^10^ As a result, some studies have found a strong link between FOF and physical-functional fitness measures such as strength, gait speed, and balance in different and complementary ways.^11^

Despite the findings discussed above, the understanding of FOF, especially when related to physiological factors, has yet to be thoroughly investigated. The association between FOF and salivary biomarkers of stress, such as Cortisol (COR), Testosterone (TT), Dehydroepiandrosterone (DHEA), and α-Amylase Activities (α-AMY), for example, has not been consistently explored. The stress-induced higher COR levels deeply influence the general health cognition.^12^ Results of recent findings suggest that the circadian COR cycle (through the hypothalamic-pituitary-adrenal axis), could also interfere with the main stress-related systems during the day.^13^ The prefrontal cortex, which is known for its importance in decision-making and attention status related to executive functions, may also be affected.^14^

The importance of several sensory-motor skills, such as decision-making and attention, and their influence on fall risk have been explored in some studies.^15^ However, in addition to the influence of COR, α-AMY is a contemporary biomarker that is used to measure psychosocial and biological stress,^16^ and indirectly could reflect attentional demand, postural control,^17^ physiological, and neuromotor changes.^18^

Another study identified that high levels of COR were associated with falls and fractures in the older adult population, with COR also being characterized as an independent predictor for hip bone fracture.^19^ Other hormones, such as TT, also showed a relationship with falls,^20^ primarily related to TT's influence on functional fitness and body composition,^21^ and showing influence on muscle strength and resistance.^22^ Moreover, the adrenal steroid DHEA showed effects on fear conditions, apparently producing the same effect/pattern as in adrenalectomy cases.^23^ In this sense, DHEA's action can influence not only fear conditions, but also mediate memory and learn processes.^24^ A recent study identified that high levels of DHEA were associated with a lower fall risk and a reduced rate of recurrent falls in the older population, with greater influence in women.^25^

A better understanding of the relationships between FOF, functional fitness, and salivary stress-related biomarkers may help deepen insight into the most relevant contributing factors and support the development of more targeted and effective interventions. The purpose of this study was to explore the association between FOF and physical-functional status, as well as salivary-related stress markers in institutionalized older women. The authors hypothesized that older women with higher levels of FOF present lower functional fitness associated with reduced salivary levels of TT and DHEA, but higher COR.

## Methods

### Study design and settings

This study was characterized as a prospective cross-sectional study involving older adults who live in Social and Health Care Centers (SHC),^26^ and followed the STROBE (Strengthening the Reporting of Observational studies in Epidemiology) statement.^27^

### Sample selection criteria

The participants were selected using a non-probability convenience sample based on the geographical area of Coimbra-Portugal, from May of 2019 to April of 2020, and specific inclusion criteria were applied: i) Women; ii) Aged over 60-years; iii) Living in the SHC; iv) Clinical condition/drug therapy controlled according to medical information; and v) Take part in the study spontaneously. Exclusion criteria were also applied: i) Presence of any type of health condition (i.e., severe cardiopathy, hypertension, uncontrolled asthmatic bronchitis, musculoskeletal conditions) that might prevent testing, according to medical decision; ii) Severe cognitive impairment (mini-mental state exam score of 9 and lower) or clinical diagnosed with mental illness; iii) Morbid obesity (body mass index of ≥ 40).

### Sample calculation

**Fig. 1:**
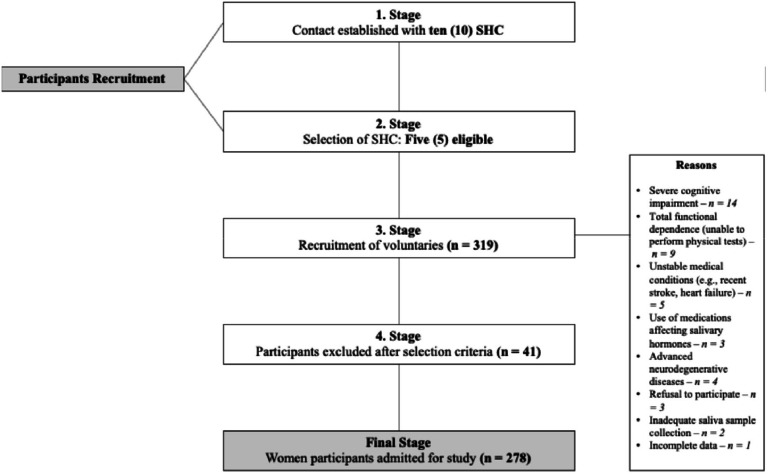
Schematic diagram of study sample flow. SHC, Social and Healthcare Centers.

An analysis of the statistical power of this study was performed and the power was determined to be 0.99. A total of 319 older women (≥ 60-years-old) were initially assessed for eligibility. After applying exclusion criteria, 41 individuals were excluded, resulting in a final sample of 278 participants included in the analyses (Fig. 1).

### Ethical aspects

The study was approved by the Ethics Committee of the Faculty of Sport Science and Physical Education, University of Coimbra (Reference: CE/FCDEF-UC/002,082,018), and conducted in accordance with the Portuguese Resolution (Art. 4; Law n° 12/2005, 1st series) and the principles of the Declaration of Helsinki.^28^ Consent forms were distributed and signed by all participants and, when applicable, their legal representatives, as well as by the directors of the residential care institutions. All procedures were carried out with institutional collaboration to ensure that participants and/or their proxies clearly understood the study objectives, procedures, and rights.

### Measures

All sample variables were encoded using a specific alphanumeric code for identification and systematically recorded. Data quality was assessed using Internal Consistency Reliability (ICR) for psychometric measures, while additional quality control methods were applied as appropriate. The Fear of Falling (FOF) was centrally analyzed. Sociodemographic, anthropometric, nutritional, and mental health variables, as well as comorbidities, were included as covariates in correlation and regression models. Functional fitness indicators, cognitive performance measures, and salivary biomarkers (e.g., cortisol, alpha-amylase) were analyzed as secondary outcomes.

### Fear of falling

The Tinetti’s Falls Efficacy Scale (FES) was used to assess FOF, where individuals rate their concern about falling during 10 daily activities on a scale from 0 to 10 for each activity.^1^ The total score ranges from 10 to 100 points, with lower scores indicating higher confidence and less FOF. To categorize participants into subgroups, the authors used the modal score of our sample, which was 60 points, as the cutoff. Participants scoring above 60 were classified as having High Fear of Falling (High FES), while those scoring 60 or below were classified as having Low Fear of Falling (Low FES). This data-driven cutoff was chosen to reflect the distribution of fear levels in our specific population, given the lack of universally established thresholds for the FES in institutionalized older adults.^29^

### Neuroendocrine salivary hormones

Saliva samples were collected between 10:00 AM and 12:00 PM to reduce the influence of circadian variation. While this standardized time frame helps control for hormonal fluctuations, it may not fully capture cortisol’s typical morning peak, potentially limiting insight into its complete diurnal profile.

Collection was performed by passive drooling for 2 min into a polypropylene tube. Participants were instructed to rinse their mouths with water 20 min before sample collection to remove potential residues or secretions. Samples were stored at −20 °C until further analysis. Salivary levels of cortisol (COR; catalog#: 1–3002), total testosterone (TT; catalog#: 1–2402), and dehydroepiandrosterone (DHEA; catalog#: 1–1202) were determined using competitive Enzyme Immunoassay (ELISA) commercial kits (Salimetrics®, UK). Alpha-amylase (α-AMY) activity was analyzed via a kinetic assay (catalog#: 1–1902; Salimetrics®, UK), following the manufacturer’s instructions.

### Functional fitness status (FFS)

The Senior Fitness Test battery was used, which included: (i) Lower-body strength (30 s Chair Stand Test – 30s-CS); (ii) Upper-body strength (30 s Arm Curl Test – 30s-AC); (iii) Lower-body flexibility (Chair Sit-and-Reach Test – CSR); (iv) Upper-body flexibility (Back Stretch Test – BST); (v) Agility and dynamic balance (8-Foot Up-and-Go Test – FUG); and (vi) Aerobic endurance (2-Minute Step Test – 2m-ST). Static balance was evaluated using the Tandem Stance Balance Test (TSB), where participants stood with one foot in front of the other, eyes open, for up to 30 s. A score of ≤ 10 s indicated poor static balance. Each test was performed three times, with the best result used for analysis.^30^ All physical assessments were conducted by trained professionals with standardized procedures. Inter-rater agreement was confirmed during preliminary calibration (ICC > 0.85).

### Sociodemographic information

This portion of data included chronological age (continuous variable), marital status (single, married, widowed, or divorced), and educational level (continuous variable), collected via questionnaire.

### Anthropometric indexes

Body weight (kg) was measured using a portable scale (Seca® 770, Germany; precision: 0.1 kg), and height (m) was assessed with a portable stadiometer (Seca Bodymeter® 208, Germany; precision: 0.1 cm). Body Mass Index (BMI; kg/m^2^) was calculated as BMI = weight/height.

### Comorbidities, medication use and nutrition

The Charlson Comorbidity Index (CCI) was used to assess comorbidities, generating a single continuous index based on 19 conditions, adjusted for age and gender. Medication use was evaluated by asking participants whether they took more or fewer than three prescription drugs daily. Polypharmacy was classified according to the Portuguese Classification System of Human Medicine. Nutritional status was assessed using the Mini Nutritional Assessment (MNA), an 18-item tool with a maximum score of 30. Participants were categorized as well-nourished (MNA ≥ 24), at risk of malnutrition (17 ≤ MNA < 24), or malnourished (MNA < 17).^26^

### Mental health assessment

Cognitive function was evaluated using the Mini-Mental State Examination (MMSE), which assesses orientation, recall, attention, calculation, delayed recall, and language (max. score: 30). Scores below 24 indicate potential cognitive impairment. Depressive symptoms were assessed using the Center for Epidemiologic Studies Depression Scale (CES-D), a 20-item questionnaire measuring symptom frequency over the past week. Responses range from 0 to 3, with total scores from 0 to 60. A cut-off of ≥16 indicates significant depressive symptoms.^26^

### Statistical analysis

Normality was assessed using the Shapiro-Wilk test and visual inspection of plots. Continuous variables were expressed as mean ± standard deviation. Fear of Falling (FOF), assessed by the Falls Efficacy Scale (FES), was considered the dependent variable. Comparisons between FOF subgroups were performed using the Student’s *t*-test or Mann-Whitney *U* test, depending on normality assumptions. Effect sizes (Cohen’s *d*) were interpreted as: trivial (< 0.20), small (0.20–0.59), moderate (0.60–1.19), large (1.20–1.99), very large (2.0–3.9), and extremely large (> 4.0).

Spearman’s rank correlation was used to assess associations between FOF, salivary biomarkers, and functional fitness status, adjusting for covariates with significant differences in subgroup comparisons. Variables that remained significantly associated with FOF after partial correlation adjustments were entered into a hierarchical stepwise multivariate regression model to assess their relative contribution to the explained variance in FOF. Correlation strength was classified as robust (*r* = 0.7–0.8), strong (*r* = 0.5–0.7), moderate (*r* = 0.3–0.4), small (*r* = 0.1–0.2), or trivial (*r* < 0.1). To account for the potential inflation of Type I error due to multiple comparisons, Bonferroni and Benjamini-Hochberg (FDR) corrections were applied to the p-values derived from the regression analyses. Statistical analyses were conducted using R 3.3.1 and IBM SPSS 22.0, with significance set at *p* < 0.05.

## Results

### Participant characteristics

As shown in Table 1, significant differences were observed between groups. The High FOF group had higher CES-D scores (*p* = 0.01, ES = 0.61), ICC scores (*p* = 0.05, ES = 0.35), and FOF scale scores (*p* < 0.001, ES = 3.10). Regarding functional fitness, participants in the Low FOF group performed better than those in the High FOF group in the 30s-CS (*p* = 0.05, ES = 0.46), 30s-AC (*p* = 0.04, ES = 0.52), 2m-ST (*p* = 0.05, ES = 0.38), TSB (*p* = 0.03, ES = 0.39), and FUG (*p* < 0.001, ES = 0.54). For salivary biomarkers, Cortisol (COR) levels were significantly higher in the High FOF group (*p* = 0.01, ES = 1.16), while DHEA levels were lower (*p* = 0.03, ES = 0.50). No significant differences were found in TT and α-AMY levels between groups.

**Table 1:** Characterization of participants and comparison analysis by fear of falling subgroups for all studied variables.

	Total sample (*n* = 278)		Higher Fear of falling (*n* = 126)		Lower Fear of falling (*n* = 152)		p-value	*Cohen’s d ES*
	M	SD	M	SD	M	SD		
**Sociodemographic**								
Chronological age (years)	81.9	7.9	82.6	7.3	81.5	8.29	0.45	0.14
Education (years)	3.6	2.7	3.5	2.8	3.7	3.05	0.66	0.08
**Anthropometric**								
Weight (kg)	65.4	12.6	66.4	14.1	64.8	11.61	0.52	0.12
Height (m)	1.5	0.0	1.5	0.0	1.5	0.08	0.17	0.28
Body mass index (kg/m^2^)	28.4	5.0	29.3	5.8	27.9	4.48	0.16	0.27
**General Health Status**								
Medication use per day (unit)	3.1	1.4	3.0	1.3	3.1	1.5	0.91	0.01
Charlson Comorbidity Index (0‒10 points)	7.4	1.8	8.8	1.5	7.2	1.9	**0.05**	0.35
Depression of CES-D (0–60 points)	21.9	8.0	24.8	8.5	20.0	7.1	**0.01**	0.61
Mini-nutritional assessment (0‒30 points)	24.3	2.3	23.5	2.5	24.8	2.1	0.12	0.13
Falls efficacy Scale (0‒100 points)	41.2	25.5	68.1	16.0	24.1	12.1	<0.001	3.10
**Functional Fitness**								
8-foot-up and go test	16.6	10.8	13.5	6.4	18.9	12.5	<0.001	0.54
Chair Seated and Reach (centimeters)	34.8	14.5	34.4	17.7	35.1	12.2	0.68	0.04
Back Stretch Test (centimeters)	48.3	22.2	44.8	17.6	50.2	24.5	0.16	0.25
30-seconds Chair Seated and Stand (per time)	8.5	3.5	7.5	3.8	9.1	3.25	**0.05**	0.46
30-second arm curl (per time)	10.7	4.3	9.4	3.6	11.6	4.5	**0.04**	0.52
2-minute step test (per time)	34.7	15.4	31.2	15.1	36.9	15.4	**0.05**	0.38
Tandem Stance Balance (seconds)	4.1	7.1	2.5	4.7	5.1	8.18	**0.03**	0.39
**Salivary biomarkers**								
Cortisol (µg/mL)	0.3	0.1	0.3	0.1	0.2	0.1	**0.01**	1.16
Dehydroepiandrosterone (pg/mL)	32.1	11.5	24.5	15.4	37.8	34.1	**0.03**	0.50
Testosterone (pg/mL)	65.2	29.6	61.8	27.9	65.4	30.6	0.32	0.12
α-Amylase (U/mL)	60.2	40.2	56.4	39.4	62.7	81.1	0.67	0.10

M, Mean; SD, Standard Deviation; ES, Effect Size; CES-D, Center for Epidemiologic Studies for Depression.

Notes: Depending on the data assumptions, the Student’s or Mann-Whitney-*U* test was used to compare Fear of Falling Subgroups.

**Fig. 2:**
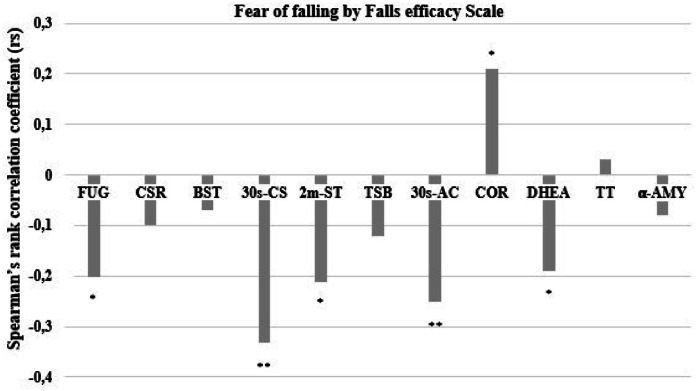
Spearman’s Ranking Correlation among fear of falling by Falls Efficacy Scale, functional fitness, and salivary biomarkers (*n* = 278). FUG, Eight Foot-Up and Go test; CSR, Chair Seated and Reach; BST, Back Stretch Test; 30s-CS, 30-seconds Chair Seated and Stand; 2m-ST, Two-minute Step Test; TSB, Tandem Stance Balance; 30s-AC, 30 s Arm-Curl test; COR, Cortisol; DHEA, Dehydroepiandrosterone; TT, Testosterone; α-AMY, α-Amylase. Significant for ** *p* ≤ 0.01; * *p* ≤ 0.05.

As the FES was the central outcome and dependent variable, Fig. 2 presents the Spearman rank correlation coefficients from the overall correlation matrix. For functional fitness, the FOF scale showed significant but small negative correlations with agility/dynamic balance (FUG: *r* = −0.202, *p* = 0.029), aerobic endurance (2m-ST: *r* = −0.212, *p* < 0.05), and upper body strength (30s-AC: *r* = −0.260, *p* < 0.01), and a moderate negative correlation with lower body strength (30s-CS: *r* = −0.331, *p* < 0.001). Regarding salivary biomarkers, FOF exhibited a small negative correlation with DHEA (*r* = −0.365, *p* = 0.033) and a small positive correlation with COR (*r* = 0.201, *p* = 0.031). No significant correlations were observed for other variables. These associations, although statistically significant, are of low-to-moderate magnitude and should be interpreted with caution regarding their practical implications.

Table 2 presents the results of the multiple linear regression analysis. Initially, multivariate models were constructed using a stepwise backward approach. Multicollinearity was assessed using the Variance Inflation Factor (VIF); all predictors showed acceptable values (VIF < 2.0). All functional fitness variables (FUG, 30s-CS, 2m-ST, 30s-AC) that showed significant correlations with FOF were included in the first analysis. This procedure yielded two statistically significant models. The model including FUG (β = −0.442; *t* = 9.148; *p* *=* 0.012; 95 % CI: −0.842 to −0.002) and 30s-CS (β = −1.664; *t* = 1.192; *p* *<* 0.001; 95 % CI: −2.952 to −0.376) demonstrated the best fit, explaining 13 % of the variance in FOF (F(2114) = 5.365; R^2^ = 0.131; *p* *<* 0.001).

**Table 2:** Multiple linear regression analysis among fear of falling, functional fitness and salivary biomarkers (*n* = 278).

	Fear of falling^a^								
	Unadjusted model 1			Adjusted model 2^b^			Adjusted model 3^c^		
	R^2^	β	p	R^2^	β	P	R^2^	β	p
**Block 1**									
30 s chair seated-and-stand	0.06	−1.690	0.012	0.16	−0.907	0.175	0.09	−0.213	0.021
**Block 2**									
30 s chair seated-and-stand	0.13	−0.178	0.049	0.19	−0.928	0.163	0.12	−0.210	0.021
Eight- foot-up-and-go		−0.442	0.012		−0.294	0.164		−0.179	0.047

a Falls efficacy scale.

b Adjusted model 2 controlling for mini-nutritional status, and state of depression.

c Adjusted model 3 controlling for cortisol and dehydroepiandrosterone.

In Model 2, MNA and CES-D were added as covariates. The model remained statistically significant (*p* < 0.001), but changes were observed: only CES-D remained statistically associated with FOF, while the effects of FUG and 30s-CS lost significance. In Model 3, salivary COR and DHEA were introduced as additional covariates. This inclusion did not affect the overall significance of the model (*p* < 0.001), but DHEA presented a stronger statistical association with FOF than other variables in this model.

**Fig. 3:**
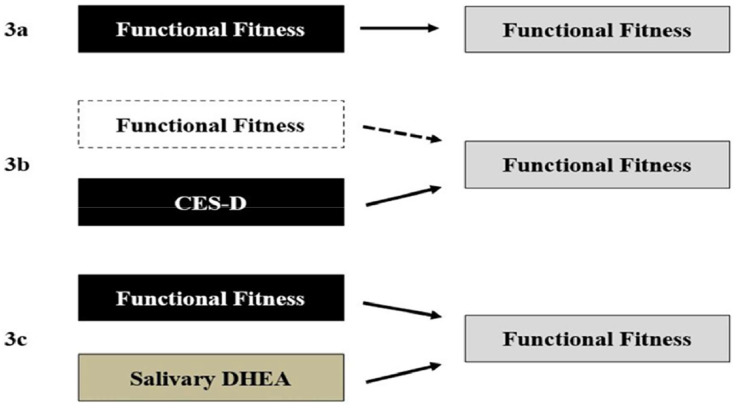
Graphical representation of multiple regression analysis (*n* = 278). (3a) Functional fitness variables predict variation in fear of falling independently. (3b) The insertion of covariates explains the impact of depressive state, which reduces the effect of functional fitness for insignificant values. (3c) The addition of salivary biomarkers as covariates had no effect on the predictive power of functional fitness indicators. On the other hand, DHEA emerges as a contributing indicator to explain the FOF variation. CES-D, Center for Epidemiologic Studies for Depression; DHEA, Dehydroepiandrosterone.

Fig. 3 presents a schematic summary of the multiple regression models, illustrating the observed statistical associations among the investigated variables. In Model 1 (3a), functional fitness indicators were significantly associated with variation in FOF. In Model 2 (3b), the inclusion of covariates ‒ specifically depressive symptoms and nutritional status ‒ was associated with a reduction in the strength of the associations between physical fitness and FOF. In Model 3 (3c), the addition of salivary biomarkers did not substantially change the model’s overall significance; however, DHEA levels were statistically associated with FOF within this model.

## Discussion

This study investigated the associations between Fear of Falling (FOF), assessed by the Falls Efficacy Scale (FES), and general health indicators (medication use, CCI, CES-D, MNA, MNES), physical-functional fitness (30s-ES, 30s-AC, CSR, BST, FUG, and 2m-ST), and salivary biomarkers of stress (TT, DHEA, COR, α-AMY) in institutionalized older women. The findings indicated that individuals with higher FOF exhibited poorer general health status, reduced functional fitness, and altered levels of stress-related salivary biomarkers.

Notably, physical-functional fitness variables were significantly associated with FOF, and regression analysis suggested that these variables accounted for part of the observed variation in FOF. When adjusting for covariates, depressive symptoms attenuated these associations, while DHEA levels remained significantly related to FOF. To our knowledge, while previous studies have examined functional, psychological, or biochemical factors related to FOF separately, this study adds value by simultaneously investigating these dimensions in institutionalized older women, offering a more integrated perspective on the multifactorial nature of FOF.

### Subgroups comparison

The initial hypothesis was partially confirmed, as significant differences were found between the groups categorized by FOF ‒ Higher FOF and Lower FOF ‒ in general health indicators, physical-functional fitness, and salivary stress biomarkers. These results are consistent with previous studies that reported differences in health-related quality of life and falls-related self-efficacy.^31^^,^^32^ Our study corroborates these findings by employing similar control variables and reaching comparable conclusions, even without considering fall history in the analysis. One previous study included both fallers and non-fallers and assessed FOF using a dichotomous question^31^; however, it did not compare physical or psychological characteristics in detail, as done here and in other works.^32^ Still, both studies pointed to independent associations between FOF and several health-related outcomes, reinforcing its relevance to quality of life and physical functioning in older adults.

Other studies have also linked physical-functional fitness, daily living activities, and quality-of-life indicators (e.g., SF-36, HRQOL) with salivary biomarkers such as DHEA and COR.^33^ Evidence suggests that older adults with lower FOF tend to demonstrate better physical performance, higher levels of DHEA, and lower levels of COR compared to those with higher FOF.^34^ Our findings support these associations, revealing that variations in general health status, functional fitness, and salivary biomarkers are related to FOF in institutionalized older women.

In line with recent meta-analyses,^35^ structured interventions focusing on social engagement and regular physical activity may help attenuate the negative effects of stress-related biomarkers (e.g., COR), muscle quality and strength indicators, and global physical-functional capacity (e.g., FUG, 30s-CS, 2m-ST). Importantly, beyond the differences observed between subgroups, the authors also identified small but significant associations across variables, underscoring the multifactorial nature of FOF.

### Associations

The authors examined the relationships among all variables and identified significant negative associations of small to moderate magnitude between FOF and physical-functional fitness measures, including FUG, 30s-CS, 2m-ST, and 30s-AC. Furthermore, FOF exhibited small but significant associations with salivary biomarkers, showing a small positive correlation with COR and a small negative correlation with DHEA. The modest strength of these correlations likely reflects the multifactorial nature of FOF, which arises from the complex interplay of physical, psychological, and physiological factors.^3^

Physical-functional fitness indicators were strongly associated with FOF. It is important to note that hormones like DHEA and COR influence physical performance, and vice versa,^36^ with COR also impacting psychological and physical condition outcomes.^18^ Both DHEA and Cortisol (COR) have been implicated in Fear of Falling (FOF), with previous studies reporting opposing effects. In our study, FOF was positively associated with COR and negatively associated with DHEA, aligning with earlier findings.^37^ Notably, Ohlsson et al. demonstrated that higher serum DHEA levels were linked to a lower risk of falls in older men, independent of physical performance metrics, suggesting that adrenal steroids may influence fall-related perceptions through non-muscular pathways. This supports our interpretation that salivary DHEA may reflect a broader neuroendocrine mechanism underlying FOF, beyond physical and psychological factors.

Similarly, another study reported a protective association between higher serum DHEA-S levels and reduced risk of falls and recurrent falls in community-dwelling older adults, particularly in women.^38^ This sex-specific effect highlights the importance of considering hormonal influences in fall-related fear and risk, especially in older female populations such as our institutionalized sample. Their findings support the notion that DHEA-S may modulate neuromuscular and psychological factors that influence fall risk, aligning with our multidimensional perspective on FOF.

Furthermore, elevated COR levels were associated with poorer physical performance in older adults,^39^ with sex-specific patterns affecting balance and strength components differently. This aligns with our finding of a positive correlation between cortisol and FOF, suggesting that heightened stress hormone levels may impair physical function and exacerbate fear of falling.

However, no significant correlations were found between FOF and TT or α-AMY. Although the Lower FOF group displayed better levels of these biomarkers compared to the Higher FOF group, the differences were not statistically significant. While TT levels have been directly linked to physical-functional fitness performance in some studies,^40^ no significant correlation with FOF was found in our sample. This is supported by the low and nonsignificant correlation coefficients between FOF and both TT and α-AMY (see Fig. 2 for an overview).

Taken together, the observed associations between FOF, physical-functional fitness, and salivary biomarkers were mostly small to moderate in magnitude. While statistically significant, these associations should be interpreted with caution, as FOF is a complex and multifactorial construct influenced by a wide range of physical, psychological, and contextual factors. The findings offer valuable insights but do not imply causal relationships, underscoring the need for longitudinal and interventional studies to better understand the directionality and practical relevance of these associations.

### Regression analysis

Our second hypothesis was partially supported by the significant results from the multiple linear regression analysis. All tests, except for TT and α-AMY salivary levels and the stretch tests (CSR, BST), showed significant associations with FOF. Specifically, better performance in physical-functional fitness tests, such as FUG, 30s-CS, 30s-AC, and 2m-ST, was related to a lower likelihood of reporting FOF, indicating a potential protective relationship with FOF.

In the older adult population, physical capabilities like gait speed, balance, and muscle strength are critical for maintaining independence.^2^ These factors are essential for stability and have a direct influence on FOF. Stability refers to the ability to return to balance after disturbances.^5^ Understanding the role of gait speed and balance is crucial for assessing fall risk and its impact on FOF, as these factors are closely interconnected. Muscle weakness, particularly in the lower limbs, can exacerbate postural instability, increasing the risk of falls and, consequently, FOF.^7^ While muscle strength is a key determinant of body balance and stability, it is important to recognize that postural instability, fall risk, and FOF are multidimensional.^35^

The pattern observed for the salivary DHEA supports this notion, as higher DHEA levels were linked to a lower likelihood of FOF, suggesting a protective effect. Previous studies have associated DHEA with improvements in muscle strength and physical performance.^33^ Conversely, although COR levels were not significantly associated with FOF in our regression analysis, other studies have found that elevated COR levels negatively affect physical performance in older adults, particularly in balance.^41^ This aligns with our finding that while COR was positively associated with FOF, physical-functional fitness indicators were negatively associated with it.

Although CES-D (depression) and MNA (nutrition) did not show direct correlations with FOF, significant differences were observed between groups. In the regression model, CES-D (depression) demonstrated a significant association with FOF. Recent research emphasizes the role of psychological and nutritional factors in the physical and physiological health of older adults. This relationship, especially concerning body balance, fall risk, and FOF, warrants further investigation.^4^

Finally, it is important to note that the regression analyses were conducted using a data-driven modeling approach,^42^ whereby only variables that showed statistically significant differences between FOF groups were included as covariates. This strategy was chosen to maintain model parsimony, minimize the risk of overfitting, and preserve statistical power. Nonetheless, the authors acknowledge that well-established factors such as physical activity, fall history, and cognitive performance are highly relevant in the FOF literature.^4^

### Potential mediating effects

Although the regression models accounted for only a modest proportion of the variance in fear of falling (9 %–19 %), this finding aligns with existing literature that conceptualizes FOF as a complex and multifactorial phenomenon. The FOF is shaped by a broad constellation of factors, including physical function, emotional well-being, cognitive status, previous fall experiences, and environmental context. Thus, it is expected that models limited to a few domains ‒ such as physical fitness, mood, and hormonal biomarkers ‒ would explain only part of the variability observed in FOF.

Furthermore, our findings reinforce the idea that FOF is not solely determined by physical performance.^5^ While functional fitness indicators showed significant associations with FOF, these relationships were attenuated when depressive symptoms and salivary biomarkers were introduced into the models. This suggests that emotional and physiological factors,^10^^,^^17^ such as mood disturbances and hormonal imbalances (e.g., reduced DHEA levels), may mediate the impact of physical limitations on perceived fall risk.^23^ In this context, depressive symptoms appear to influence how older adults perceive their functional capacity, potentially amplifying their vulnerability. The modest but consistent contribution of DHEA also indicates a potential neuroendocrine mechanism in the regulation of fear and stress responses.

### Strengths, limitations, and future directions

A key strength of this study lies in its comprehensive examination of the relationship between FOF and salivary biomarkers, such as COR and DHEA, in institutionalized older women. Through the integration of assessments of general health, functional fitness, and stress biomarkers, the research adds important insights into factors associated with FOF, highlighting significant links with COR and DHEA levels, alongside the influence of functional fitness and depressive symptoms.

However, there are several limitations. The cross-sectional design limits the ability to infer causal relationships or changes over time. The relatively small sample size, combined with the use of a non-probability convenience sample from a single geographic region, may limit the generalizability of the findings. Although only 41 participants (12.9 %) were excluded, the potential for selection bias should also be acknowledged, reinforcing the need for larger and more representative studies. Another important limitation is the use of single-time point saliva sampling, which restricts the reliability of estimating chronic endocrine status, given the natural diurnal variability of biomarkers like cortisol and DHEA. Future research should incorporate repeated saliva measurements across different times and days to better capture hormonal fluctuations and improve reliability.

This study focused exclusively on institutionalized older women, a population with increasing global relevance due to rising long-term care residency rates. However, this focus limits the generalizability of our findings to other subgroups, such as community-dwelling older adults or men, who may present distinct physiological or psychological characteristics related to FOF. Overall, our results provide important insights into the mechanisms underlying FOF in older adults and suggest directions for future research, particularly in exploring biological markers to enhance autonomy and independence.

### Real-world practical applications

This study's findings suggest practical applications for improving fall prevention and managing Fear of Falling (FOF) in older adults. Personalized fall prevention programs could be developed by combining functional fitness interventions, such as balance and strength exercises, with stress-reduction strategies tailored to individual biomarker profiles (e.g., COR, DHEA). A holistic approach addressing both physical and mental health ‒ through depression screenings and targeted fitness routines ‒ can further reduce FOF.

Monitoring salivary biomarkers regularly could serve as an early warning system for fall risk, enabling timely interventions. Additionally, wearable technology that tracks physical activity and stress markers could provide real-time data to prevent falls. In institutional settings, integrating physical exercises with psychological and nutritional support, such as DHEA-boosting foods, can help reduce FOF and improve overall independence. These approaches highlight the importance of combining physical, psychological, and biological factors in fall prevention strategies for older adults.

## Conclusion

This study identified significant associations between physical performance, salivary hormone levels, and FOF, indicating that these variables are related to variations in FOF. The results emphasize the importance of incorporating physical-functional fitness and stress biomarkers like COR and DHEA in understanding and addressing FOF in older adults. These findings offer valuable insights for health professionals, guiding the development of tailored intervention programs aimed at preventing or minimizing FOF. Such programs could effectively reduce the incidence of falls and mitigate their associated risks and consequences, promoting overall well-being and independence in older populations.

## Availability of data and materials

The datasets used and analyzed during the current study are available from the corresponding author upon reasonable request.

## Funding

This work was partially financed by FEDER funds through COMPETE and national funds through FCT ‒ Portuguese Foundation for Science and Technology in the framework of the project (PTDC/DTPDES/0154/2012).

## CRediT authorship contribution statement

**Rafael N. Rodrigues:** Conceptualization, Methodology, Formal analysis, Writing – original draft, Writing – review & editing. **Dina Maria Mamede Pereira:** Conceptualization, Methodology, Supervision, Writing – review & editing. **Sónia Brito-Costa:** Formal analysis, Writing – original draft, Writing – review & editing. **António Felipe Souza-Gomes:** Investigation, Methodology, Writing – review & editing. **Natália Oiring de Castro Cezar:** Investigation, Writing – review & editing. **Júlia Maria D’Andréa Greve:** Methodology, Writing – review & editing. **Angelica Castilho Alonso:** Conceptualization, Writing – review & editing. **Felipe Santos Marques:** Writing – review & editing. **José Pedro Ferreira:** Investigation, Writing – review & editing. **Ana Maria Teixeira:** Methodology, Supervision, Writing – review & editing. **Guilherme Eustáquio Furtado:** Conceptualization, Supervision, Writing – review & editing.

## Declaration of competing interest

The authors declare no conflicts of interest.
